# The full management from first-line to third-line treatments in patients with Her-2–negative advanced gastric cancer

**DOI:** 10.3389/fonc.2022.949941

**Published:** 2022-11-15

**Authors:** Chunxiao Chang, Yanqing Pei, Jun Xu, Wenyu Zhang, Jianbo Zhang, Shengbin Shi

**Affiliations:** ^1^ Department of Gastrointestinal Oncology, Shandong Cancer Hospital and Institute, Shandong First Medical University and Shandong Academy of Medical Sciences, Jinan, Shandong, China; ^2^ Department of Infection Management, Shandong Cancer Hospital and Institute, Shandong First Medical University and Shandong Academy of Medical Sciences, Jinan, Shandong, China; ^3^ Department of Imaging, Shandong Cancer Hospital and Institute, Shandong First Medical University and Shandong Academy of Medical Sciences, Jinan, Shandong, China; ^4^ Department of Pathology, Shandong Cancer Hospital and Institute, Shandong First Medical University and Shandong Academy of Medical Sciences, Jinan, Shandong, China

**Keywords:** gastric cancer, full management, chemotherapy, immune checkpoint inhibitors, Her-2 negative patients

## Abstract

**Background:**

The aim of this study was to retrospectively evaluate the efficacy of full management from first-line to third-line treatments in patients with human epidermal growth factor receptor 2 (Her-2)–negative advanced gastric cancer (GC).

**Methods:**

The efficacy and survival time of a total of 126 patients who received the first-line treatment with oxaliplatin plus fluoropyrimidine (S-1 or capecitabine or fluorouracil), the second-line treatment with nab-paclitaxel, and the third-line treatment of immune checkpoint inhibitors between September 2019 and December 2021 were analyzed.

**Results:**

A total of 42, 36, and 48 patients received CapeOX, FOLFOX, and SOX as a first-line treatment, respectively. All patients received nab-paclitaxel alone as a second-line treatment. In addition, 31, 56, and 39 patients received nivolumab, sintilimab, and tislelizumab as a third-line treatment, respectively. The median PFS1, median PFS2, and median PFS3 was 6.9 months [95% confidence interval (CI), 6.8–7.4], 5.5 months (95% CI, 5.3–5.7), and 3.5 months (95% CI, 3.4–3.7). The median PFS3 was 3.8 months (95% CI, 3.3–4.2) and 3.5 months (95% CI, 3.3–3.7) among the Epstein–Barr virus (EBV)-positive and EBV-negative, respectively (P = 0.09). In addition, the median PFS3 was 4.2 months (95% CI,3.6–4.7) and 3.5 months (95% CI, 3.3–3.6) in the patients with programmed death ligand 1 (PD-L1) combined positive score (CPS) ≥5 and CPS <5, respectively (P = 0.02). The median OS was 17.4 months (95% CI, 17.2–18.1). The multivariate analysis showed that the two parameters were associated with a significantly longer OS: number of metastatic sites <3 and PD-L1 CPS ≥5.

**Conclusion:**

The patients who received three lines of treatment had a long survival time, and the efficacy of immunotherapy was not affected by the EBV subtypes in advanced GC. The toxicity was managed, and the concept of full management needs to be confirmed in the future.

## Introduction

Gastric cancer (GC) is one of the most common cancers, being the third leading cause of cancer death worldwide. Moreover, it is the second leading cause of cancer death in Eastern Asia (1). Surgery is recommended as the mainstay of curative treatment for early-stage patients with GC, but most patients experience relapse after surgery with a high recurrence rate at approximately 40%–80% (2). However, approximately 50% of patients are already locally advanced or have metastasis and lose the surgical opportunity at diagnosis (3). For these patients, systemic chemotherapy is the recommended standard treatment, and the 5-year survival rate is less than 4.5% (4).

First-line chemotherapy prolonged the survival time compared with best supportive care alone in patients with locally advanced or metastatic disease (5, 6). Therefore, the European Society of Medical Oncology and the National Comprehensive Cancer Network recommend the regimen of platinum and fluoropyrimidine as a standard treatment (7). For patients with human epidermal growth factor receptor 2 (Her-2)–negative advanced GC, the median survival time was 12.0 months with treatment of the XELOX or EOX regimen (8). For patients who are Her-2–positive, the TOGA study demonstrated that trastuzumab plus platinum-based chemotherapy prolonged the survival time compared with chemotherapy alone. The median overall survival (OS) was 13.8 months compared with 11.1 months (P = 0.0046) (9).

After failure of the first-line chemotherapy, second-line palliative chemotherapy is recommended as the standard of treatment. Several review and meta-analysis demonstrated the OS advantage of second-line chemotherapy compared with best supportive care (BSC) alone with extended OS by approximately 6.7 months (2, 10, 11). Taxane-based (docetaxel and paclitaxel) and irinotecan are recommended as a standard second-line chemotherapy (12). Ramucirumab alone or combined with paclitaxel also demonstrated the superior efficacy with prolonged OS and recommended as a standard second-line treatment (13, 14). One study shows that weekly paclitaxel used as a second-line treatment revealed a median OS of 5–6 months and overall response rates (ORRs) of 16%–24% in Japan (15). In the phase III WJOG4007 trial, where paclitaxel or irinotecan was used as a second-line treatment for advanced GC, the OS did not show a significant difference between the two groups (16). One study of nab-paclitaxel versus solvent-based paclitaxel in patients with previously treated advanced GC demonstrated that weekly nab-paclitaxel was non-inferior to weekly solvent-based paclitaxel in terms of OS (17).

In one retrospective trial of a single institutional experience, 27% of advanced GC patients received a third-line treatment (18). Many patients have a good general performance after failing the second-line treatment. Evidence shows that third-line or later lines of treatment prolonged the OS in patients with advanced GC. Third-line drugs include irinotecan, taxane, trifluridine/tipiracil (TAS-102), apatinib, and immune checkpoint inhibitors. One phase III randomized trial demonstrated that nivolumab significantly prolonged the OS compared to placebo (19). The median OS of third-line treatment was less 5.0 months. However, there is no report on the efficacy of the first-line to third-line treatments in advanced GC. In this study, we retrospectively evaluated the treatment regimen of oxaliplatin plus oral fluoropyrimidine (S-1 or capecitabine or fluorouracil) in the first line, of nab-paclitaxel in the second line, and immune checkpoint inhibitors in the third line in patients with Her-2–negative advanced GC.

## Patients and methods

### Patients

In this study, we retrospectively analyzed patients with Her-2–negative metastatic GC. Patients were diagnosed with gastric or gastroesophageal junction adenocarcinoma, who were histologically confirmed at Shandong Cancer Hospital and Institute from September 2019 to December 2021. This study was conducted with the approval of the Shandong Province Tumor Hospital Ethics Committee (2022004008). The eligibility criteria are as follows: older than 18 years, performance status (PS) of 0–2, adequate organ function, no serious clinical complications, no measurable central nervous system metastasis, and evaluable lesions according to the Response Evaluation Criteria in Solid Tumors (RECIST) (version 1.1) criteria; patients were treated with the first-line therapy of oxaliplatin plus fluoropyrimidine (S-1 or capecitabine or fluorouracil), the second-line therapy of nab-paclitaxel, and the third-line therapy of immune checkpoint inhibitors.

### Treatment and evaluation of response

Patients were treated with first-line SOX (oxaliplatin at 130 mg/m^2^ on day 1 plus oral S-1 at 40 mg/m^2^ twice a day, on days 1–14, every 3 weeks), CapeOX (oxaliplatin at 130 mg/m^2^ on day 1 plus capecitabine at 1,000 mg/m^2^ twice a day, on days 1–14, every 3 weeks), or FOLFOX (fluorouracil at 400 mg/m² on day 1 and at 1,200 mg/m² on days 1–2, oxaliplatin at 85 mg/m² on day 1, and leucovorin at 400 mg/m² on day 1, every 2 weeks) for up to 6 months, and after that, patients without evidence of disease progression received maintenance treatment with S-1 or capecitabine or fluorouracil. A second-line treatment with nab-paclitaxel at 100 mg/m² was administered weekly (on days 1, 8, and 15) every 4 weeks and was continued without limitation of maximum treatment cycles until disease progression and occurrence of unacceptable severe toxicity. The third-line of immune checkpoint inhibitors that were a third-line treatment (nivolumab, 3 mg/kg; sintilimab, 200 mg; and tislelizumab 200 mg, intravenously) was administered every 3 weeks alone. A third-line treatment was given until disease progression, occurrence of unacceptable toxicity, and up to a maximum of 2 years. Toxicity evaluations were evaluated by the National Cancer Institute’s Common Terminology Criteria for Adverse Events, version 4.0. The dose reductions were decided on the basis of the patient’s general condition and the hematological and nonhematological toxicity. Tumor response was evaluated by computerized tomography images every two or three cycles and was classified by the standard RECIST, version 1.1.

### Statistical analysis

Progression-free survival (PFS) was calculated from the first day of each regimen, respectively, to the date of disease progression (PFS1 was calculated from the first day of the first-line treatment regimen to the date of disease progression; PFS2 was calculated from the first day of the second-line treatment regimen to the date of disease progression; and PFS3 was calculated from the first day of the third-line treatment regimen to the date of disease progression). OS was defined as the date of initiation of first-line chemotherapy to the time of death. The Kaplan–Meier method was used to calculate OS and PFS. Descriptive statistics were presented with percentage and medians. Statistical analyses were calculated using SPSS software (version 18.0, SPSS Inc., Chicago, IL, USA), and P-values less than 0.05 were considered significant.

## Results

### Patient characteristics

In this study, we retrospectively analyzed 352 patients with Her-2–negative metastatic GC, and 126 patients were included in this retrospective study; the patient characteristics are presented in **Table 1**. The median age was 57 years (range, 37–78), and the primary tumor site included the stomach (76.9%) and gastroesophageal junction (23.1%). Eighty patients (69.0%) were presented in the Eastern Cooperative Oncology Group (ECOG) PS of 0–1. The metastasis mainly included the distant lymph nodes (41.3%), liver (36.5%), and peritoneum (22.2%). In addition, 25 patients (19.8%) underwent PD-L1 combined positive score (CPS) ≥5, and 19 patients (15.1%) showed EBV positivity. In these patients, 42 (33.3%), 36 (28.6%), and 48 patients (38.1%) received CapeOX, FOLFOX, and SOX regimens, respectively, as a first-line treatment. All patients received nab-paclitaxel alone as a second-line treatment. In addition, 31 (24.6%), 56 (44.4%), and 39 patients (31.0%) received nivolumab, sintilimab, and tislelizumab, respectively, as a third-line treatment.

**Table 1:** Patient characteristics.

Characteristic	n (%)
Age, years	
Median age (range)	57 (37–78)
Sex	
Male Female	74 (58.7%) 52 (41.3%)
Performance status	
0–1 2	87 (69.0%) 39 (31.0%)
Tumor location	
Gastric Gastroesophageal junction	97 (76.9%) 29 (23.1%)
Metastatic sites	
Liver Peritoneum Lymph node	46 (36.5%) 28 (22.2%) 52 (41.3%)
PD-L1	
≥5 <5	25 (19.8%) 101 (80.2%)
EBV	
Positive Negative	19 (15.1%) 107 (84.9%)
First-line chemotherapy	
CapeOX FOLFOX SOX	42 (33.3%) 36 (28.6%) 48 (38.1%)
Time to progressive disease on first-line therapy (PFS 1)	
<6 months ≥6 months	35 (27.8%) 81 (72.2%)
Second-line chemotherapy	
Nab-paclitaxel	126
Time to progressive disease on second therapy (PFS 2)	
PFS <5 months PFS ≥5 months	49 (38.9%) 77 (61.1%)
Third-line treatment	
Nivolumab Sintilimab Tislelizumab	31 (24.6%) 56 (44.4%) 39 (31.0%)
Time to progressive disease on third therapy (PFS 3)	
PFS <2 months PFS ≥2 months	47 (37.3%) 79 (62.7%)

### Treatment administration and response

**Figure 1:**
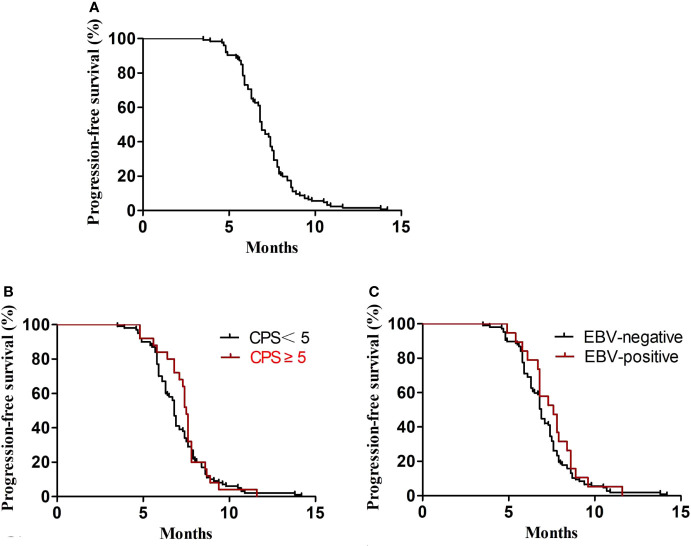
PFS1. **(A)** Kaplan–Meier plots for PFS1 in the first-line treatment. **(B)** PFS1 according to PD-L1 (P = 0.43). **(C)** PFS1 according to EBV (P = 0.31).

**Figure 2:**
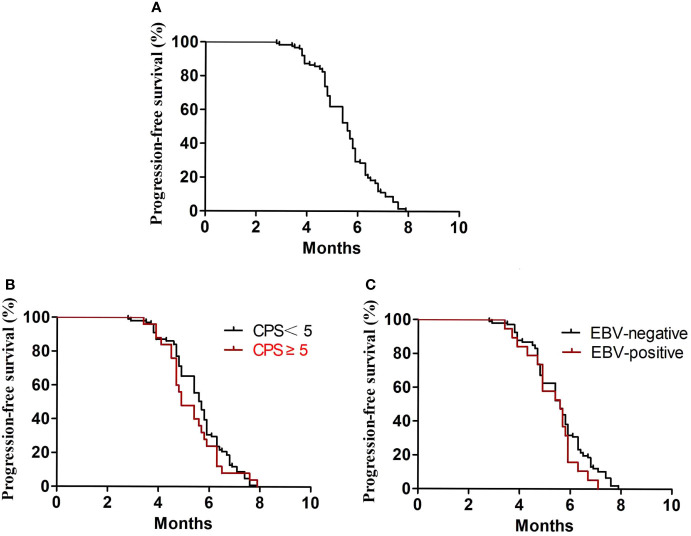
PFS2. **(A)** Kaplan–Meier plots for PFS2 in the second-line treatment. **(B)** PFS2 according to PD-L1 (P = 0.36). **(C)** PFS2 according to EBV (P = 0.46).

**Figure 3:**
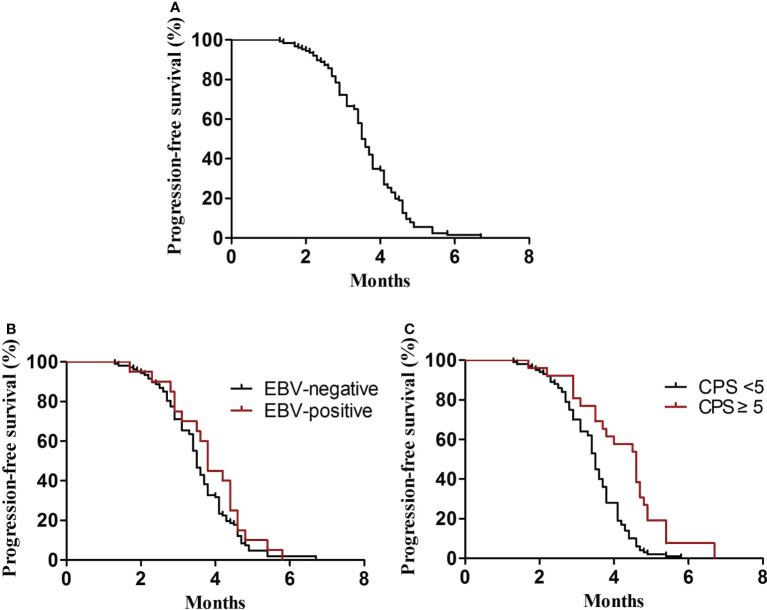
PFS3. **(A)** Kaplan–Meier plots for PFS3 in the third-line treatment. **(B)** PFS3 according to PD-L1 (P = 0.02). **(C)** PFS3 according to EBV (P = 0.09).

**Figure 4:**
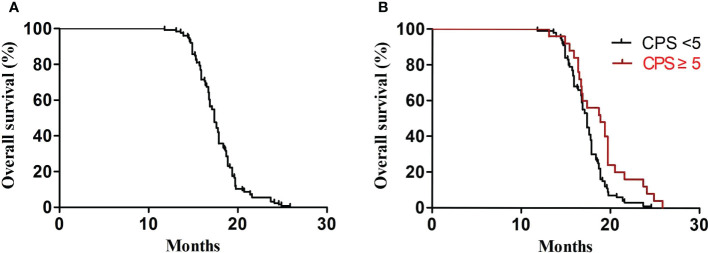
OS. **(A)** Kaplan–Meier analysis of OS in all advanced GC patients. **(B)** OS according to PD-L1 (P = 0.03).

In the first-line treatment, the ORR and disease control rate (DCR) were 39.7% and 84.1%, respectively. The median PFS1 was 6.9 months (95% CI, 6.8–7.4 months) (**Figure 1A**). The median PFS1 was 7.5 months (95% CI, 6.4–7.9 months) and 6.8 months (95% CI, 6.2–7.4 months) in the patients with PD-L1 CPS ≥5 and CPS <5, respectively (P = 0.12) (**Figure 1B**). The median PFS1 was 7.6 months (95% CI, 6.7–8.0 months) and 6.9 months (95% CI, 6.3–7.5 months) among patients who are EBV-positive and EBV-negative, respectively (P = 0.18) (**Figure 1C**). In the second-line therapy, all patients received treatment with nab-paclitaxel, and the median PFS2 was 5.5 months (95% CI, 5.3–5.7 months) (**Figure 2A**). The ORR and DCR were 29.4% and 73.1%, respectively. The median PFS2 was 5.7 months (95% CI, 5.1–6.1 months) and 4.9 months (95% CI, 4.4–5.4 months) in the patients with PD-L1 CPS ≥5 and CPS <5, respectively (P = 0.14) (**Figure 2B**). The median PFS2 was 5.6 months (95% CI, 5.3–5.9 months) and 5.6 months (95% CI, 5.2–5.8 months) among patients who are EBV-positive and EBV-negative, respectively (P = 0.53) (**Figure 2C**). For the third-line treatment with nivolumab or sintilimab or tislelizumab, no complete response was observed, and the median PFS3 was 3.5 months (95% CI, 3.4–3.7 months) (**Figure 3A**). In addition, the ORR and DCR were 11.1% and 53.2%, respectively (**Table 2**). The median PFS3 was 4.6 months (95% CI, 3.8–4.8 months) and 3.4 months (95% CI, 3.2–3.6 months) in the patients with PD-L1 CPS ≥5 and CPS <5, respectively (P = 0.02) (**Figure 3B**). In addition, the median PFS3 was 3.8 months (95% CI, 3.3–4.2 months) and 3.5 months (95% CI, 3.3–3.7 months) among patients who are EBV-positive and EBV-negative, respectively (P = 0.09) (**Figure 3C**). The median OS was 17.4 months (95% CI, 17.2–18.1 months) (**Figure 4A**), and the median OS was 18.9 months and 17.4 months in the patients with PD-L1 CPS ≥5 and CPS <5, respectively (P = 0.03) (**Figure 4B**).

**Table 2:** Efficacy of treatments.

Enrolled patients	n (%)
Tumor response on first-line therapy	
Complete response (CR) Partial response (PR) Stable disease (SD) Progressive disease (PD) Objective response rate (ORR) DCR PFS 1, median (months; 95% CI) ORR PD-L1 ≥ 5 ORR PD-L1 < 5 PFS 1 PD-L1 ≥ 5 PFS 1 PD-L1 < 5	3 (2.4%) 47 (37.3%) 56 (44.4%) 20 (15.9%) 39.7% 84.1% 6.9 (6.8–7.4) 40.0% 39.6% 7.5 (6.4–7.9) 6.8 (6.2–7.4)
Tumor response on second therapy	
CR PR SD PD ORR DCR PFS 2, median (months; 95% CI) ORR PD-L1 ≥ 5 ORR PD-L1 < 5 PFS 2 PD-L1 ≥ 5 PFS 2 PD-L1 < 5	2 (1.6%) 35 (27.8%) 55 (43.7%) 34 (26.9%) 29.4% 73.1% 5.5 (5.3–5.7) 32.0% 28.7% 5.7 (5.2–6.5) 4.9 (4.4–6.1)
Tumor response on third therapy	
CR PR SD PD ORR DCR PFS 3, median (months; 95% CI) EBV- Positive EBV- Negative PD-L1 ≥ 5 PD-L1 < 5 OS, median (months; 95% CI) PD-L1 ≥ 5 PD-L1 < 5	0 14 (11.1%) 53 (42.1%) 59 (46.8%) 11.1% 53.2% 3.5 (3.4–3.7) 3.8 (3.3–4.2) 3.5 (3.3–3.7) 4.6 (3.8–4.8) 3.4 (3.2–3.6) 17.4 (17.2–18.1) 18.9 (17.1–19.7) 17.4 (16.8–18.8)

### Prognostic factors and adverse events

For the prognostic factors, we analyzed some potential characteristics for affecting OS by multivariate analysis. There were two parameters associated with a significantly longer OS: number of metastatic sites ≤3 and PD-L1 CPS ≥5 (**Table 3**). The state of EBV did not affect the OS. The toxicity mainly included hematological and nonhematological parameters (**Table 4**). The most common grade 3–4 hematological toxicity was neutropenia (20.6%), anemia (2.4%), and thrombocytopenia (3.9%). Grade 3–4 nonhematological toxicities included nausea (3.9%), diarrhea (4.8%), and mucositis (3.9%), and fatigue (7.9%). There were no treatment-related deaths.

**Table 3:** Multivariate analysis for OS.

	HR	95% CI	p
ECOG PS (0–1 vs. 2) Age (<65 vs. ≥65 years) PFS 1 (<6 vs. ≥6 months) PFS 2 (<5 vs. ≥5 months) PFS 3 (<2 vs. ≥2 months) Metastatic sites (<3 vs. ≥3) PD-L1 (CPS <5 vs. ≥5) EBV (positive vs. negative)	0.63 1.42 0.61 0.73 1.78 4.87 3.42 1.23	0.32–1.08 0.58–2.91 0.34–1.24 0.39–1.61 0.81–3.42 2.29–6.64 2.43–5.02 0.76–3.45	0.31 0.96 0.31 0.39 0.18 0.01 0.03 0.71

HR, hazard ratio.

**Table 4:** Adverse events.

	All grades	Grade≥3
Hematologic		
Neutropenia Anemia Thrombocytopenia	40 (31.7%) 23 (18.3%) 28 (22.2%)	26 (20.6%) 3 (2.4%) 5 (3.9%)
Non-hematologic		
Nausea Vomiting Diarrhea Mucositis Asthenia Fatigue	39 (30.9%) 19 (15.1%) 14 (11.1%) 15 (11.9%) 11 (8.7%) 18 (14.3%)	5 (3.9%) 4 (3.2%) 6 (4.8%) 5 (3.9%) 3 (2.4%) 5 (3.9%)

## Discussion

For advanced GC, a standard palliative treatment is chemotherapy-based, which improves the quality of life and prolongs survival compared with best supportive care alone. Fluoropyrimidine plus platinum have been demonstrated to have survival benefits in the first-line treatment for advanced GC (20, 21). In advanced GC, Her-2 amplification or overexpression rate was shown to be approximately 7%–34% (20). Therefore, most patients are Her-2–negative, and the first line treatment of metastatic GC was platinum-based and fluoropyrimidine chemotherapy (22).

Cisplatin has been an important compound in the treatment of patients with GC for many years. With nausea, vomiting, and renal toxicity as side effects, oxaliplatin was also investigated (23) and also demonstrated a similar efficacy when compared with cisplatin (24, 25). In our study, CapeOX, SOX, or FOLFOX as a first-line treatment for metastatic GC showed an ORR of 39.7%, a DCR of 84.1%, and the median PFS of 6.9 months. In the study of first-line nivolumab plus chemotherapy versus chemotherapy alone for advanced gastric, gastro-esophageal junction, and esophageal adenocarcinoma, the administered chemotherapy regimen included capecitabine and oxaliplatin every 3 weeks or leucovorin, fluorouracil, and oxaliplatin every 2 weeks. The median PFS was 6.0 months (5.6–6.9) in the chemotherapy group alone (26), and the PFS was similar to our study.

After progression of the first-line treatment, all patients received treatment with nab-paclitaxel. One study of nab-paclitaxel versus solvent-based paclitaxel in patients

with previously treated advanced GC demonstrated that the median PFS was 5.3 months (4.0–5.6) in the weekly nab-paclitaxel group (17). In our study, the PFS was 5.5 months (5.3–5.7), which showed to be more prolonged, compared with above study. In the second-line treatment, the PFS that was affected by the first-line treatment is not known. Some randomized studies demonstrated that the second-line chemotherapy prolongs mOS to approximately 1.5 months compared with BSC alone (27–29). Second-line chemotherapy could improve the quality of life and prolong survival; however, the average benefit of mOS is at 6 weeks (16, 27, 28). Patients who have a long PFS and an excellent PS in the first-line chemotherapy receive the most benefit in the second-line treatment (30).

Most patients with progression in the second-line treatment have an opportunity to receive a third line of therapy or further chemotherapy. Some studies have demonstrated the use of chemotherapy in the third-line treatment in recent years (14, 31, 32). Nishimura et al. conducted one study where 52 patients received irinotecan monotherapy as a third-line treatment in advanced GC, and the median PFS was 2.3 months and the median OS was 4.0 months (33). With the development of immunotherapy, the third-line treatment showed the most promising results in advanced GC. Nivolumab, a human anti-programmed cell death 1 antibody, significantly prolonged the median OS and median PFS compared with the best supportive care as a third-line treatment for advanced GC (19). At present, there is no study to confirm the superior efficacy of a third-line treatment with irinotecan monotherapy or immunotherapy. Patients with MSI-high and EBV subtypes treated with immune checkpoint inhibitors have demonstrated a high efficacy (34, 35). In our study, no patients with MSI-high were included, and patients who EBV-positive did not show a superior survival in the treatment with immune checkpoint inhibitors. This could be attributed to fact that EBV causing GC only afflicts a small percentage of patients, and EBV-related carcinogenesis leads to microsatellite instability within this region (36–38). Compared with other studies, the present median OS and PFS of our study were longer, suggesting that patients who received three lines of treatment have a good prognosis. Some studies have shown that the EBV and PD-L1 expression were related to the efficacy of immunotherapy. In contrast, EBV and PD-L1 expression were not related to the efficacy of chemotherapy.

In conclusion, patients who received three lines of treatment may have a long survival time, and the efficacy of immunotherapy was not affected by the EBV subtypes. The toxicity was managed, and no patients died because of toxicity. Our study has some limitations with a retrospective analysis in a single center and a small number of patients who received full management from first-line to third-line treatments. This needs a large population of patients to confirm the efficacy of a complete management in advanced GC.

## Data availability statement

The original contributions presented in the study are included in the article/supplementary material. Further inquiries can be directed to the corresponding author.

## Ethics statement

The studies involving human participants were reviewed and approved by Shandong Province Tumor Hospital Ethics Committee (2022004008). Written informed consent from the participants’ legal guardian/next of kin was not required to participate in this study in accordance with the national legislation and the institutional requirements.

## Author contributions

CC and YP performed the experiments, analyzed the data and prepared the manuscript. JX and WZ aided the data analysis and manuscript preparation. JZ contributed data analysis. SS designed and supervised the study, analyzed the data, prepared and revised the manuscript. All authors read and approved the final manuscript.

## Funding

This research was supported by the Start-up fund of Shandong Cancer Hospital (2020-PYB11).

## Conflict of interest

The authors declare that the research was conducted in the absence of any commercial or financial relationships that could be construed as a potential conflict of interest.

## Publisher’s note

All claims expressed in this article are solely those of the authors and do not necessarily represent those of their affiliated organizations, or those of the publisher, the editors and the reviewers. Any product that may be evaluated in this article, or claim that may be made by its manufacturer, is not guaranteed or endorsed by the publisher.
